# EGFR-TKI在非小细胞肺癌中耐药机制的研究进展

**DOI:** 10.3779/j.issn.1009-3419.2013.10.07

**Published:** 2013-10-20

**Authors:** 慧慧 刘, 孟昭 王, 克 胡, 燕 徐, 满姣 马, 巍 钟, 静 赵, 龙芸 李, 华竹 王

**Affiliations:** 1 100730 北京，中国医学科学院北京协和医院呼吸内科 Department of Respiratory Medicine, Peking Union Medical College Hospital, Beijing 100730, China; 2 100730 北京，中国医学科学院北京协和医院放疗科 Department of Radiotherapy, Peking Union Medical College Hospital, Beijing 100730, China

**Keywords:** 肺肿瘤, EGFR, EGFR酪氨酸激酶抑制剂, 耐药, Lung neoplasms, EGFR, EGFR-TKIs, Resistance

## Abstract

目前，肺癌是全世界范围内发病率和死亡率最高的恶性肿瘤，其中非小细胞肺癌（non-small cell lung cancer, NSCLC）占全部肺癌的80%左右，而NSCLC患者中有很大一部分在确诊时已经处于晚期。因此，对于晚期NSCLC的治疗也越来越受到人们的重视。虽然晚期NSCLC的标准治疗为含铂双药联合化疗，但是化疗药物对改善晚期NSCLC患者的生存期方面作用十分有限，因此寻求新的治疗方式迫在眉睫。随着对肺癌发病机制及其生物学行为的深入研究，分子靶向治疗已成为治疗晚期NSCLC最具前景的研究领域。其中表皮生长因子受体-酪氨酸激酶抑制剂（epidermal growth factor receptor tyrosine kinase inhibitors, EGFR-TKIs）在晚期NSCLC治疗方面取得了突破性进展，其代表药物为吉非替尼和厄洛替尼，这两种EGFR-TKIs已在全世界范围内得到认可并被广泛用于晚期NSCLC的治疗，尤其是对于EGFR敏感突变者。然而，经过一段时间（中位时间为6个月-12个月）的治疗后，大部分患者会对EGFR-TKIs产生耐药，其耐药机制主要包括原发性和获得性耐药。由于EGFR-TKIs在改善晚期NSCLC患者总生存期和无进展生存期方面的突出作用，对于EGFR-TKIs耐药机制的探索已成为国内外研究的热点。该文章就EGFR-TKI耐药机制的研究进展进行了综述。

## Abstract (en)

Nowadays, lung cancer is the malignant tumor of the highest morbidity and mortality over the world, and non-small cell lung cancer (NSCLC) makes up about 80%. There is a great many NSCLC patients have been in advanced stage when diagnosed. As a result, people pay more attention to curing advanced NSCLC. The standard treatment to advanced NSCLC is platinum-based combined chemotherapy. However, chemotherapy drugs usually have limited effects on improving the survival of the patients. Then exploring new therapies is extremely urgent to us. Now, molecular targeted therapy has been the most promising research area for the treatment of NSCLC with researches going deep into pathogenesis and biological behavior of lung cancer. Epidermal growth factor receptor-tyrosine kinase inhibitors (EGFR-TKIs) have achieved a great success in the treatment of advanced NSCLC. Their representatives are erlotinib and gefitinib. The two drugs have been widely used to treat advanced NSCLCs worldwide, especially for the patients with EGFR activating mutations. However, after a period of treatment (median time is 6 to 12 months), most patients will develop drug resistance to EGFR-TKIs. Intense research in these NSCLCs has identified two major mechanisms of resistance to TKIs: primary and acquired resistances. The research about resistance mechanism of NSCLC to EGFR-TKIs is a hot one because of their excellent effects on improving overall and progression-free survival. The aim of this article was to summarize the development of the resistance mechanisms.

## 1 前言

肺癌中表皮生长因子受体（epidermal growth factor receptor, *EGFR*）突变主要发生在胞内段编码结构域（外显子18-21），包括外显子19的缺失突变（delE746-A750）和外显子21点突变（L858R）^[1, 2]^，两者占所有EGFR激酶突变的90%以上，与对表皮生长因子受体-酪氨酸激酶抑制剂（EGFR tyrosine kinase inhibitors, EGFR-TKIs）的敏感性有关；此外，还有外显子18点突变（G719S）以及外显子20插入突变，前者属于EGFR-TKI的敏感突变而后者与EGFR-TKI的耐药有关^[3]^，发生率均在5%左右。通常情况下，*EGFR*突变的NSCLC患者对TKI的治疗较敏感。EGFR-TKIs通过与ATP或底物竞争性结合胞外的配体结合位点，阻断EGFR分子内酪氨酸的自身磷酸化及酪氨酸激酶的活化，抑制EGFR同源或与ERBB3异源二聚体的形成，从而抑制EGFR激活，阻止下游信号转导，抑制细胞周期进程、加速细胞凋亡、抑制血管生成和转移。但是在临床工作中，许多患者对EGFR-TKI的治疗并不敏感，或者是在治疗一段时间后产生耐药。其耐药机制主要包括原发性耐药与获得性耐药。本文将总结现有对EGFR-TKI耐药机制的研究，对其现况及进展进行综述。

## 2 原发性耐药

原发性耐药是指首次使用EGFR-TKI即产生耐药，约60% NSCLC患者的耐药为TKI原发性耐药。其中，*EGFR*基因激活突变者有近30%对TKI原发耐药。

### 2.1 *KRAS*突变

KRAS是EGFR下游的一个重要信号传导通路，这两者都与肺癌的发生和治疗密切相关。突变后的*KRAS*基因不依赖于上游EGFR的活化而直接激活MAPK信号通路，导致肿瘤增殖、转移等^[4]^。大约15%-20%的NSCLC患者中存在*KRAS*突变。*KRAS*突变与吸烟有关。Eberhard等^[5]^的研究中10例非吸烟患者中均未检测到*KRAS*突变。同时，有研究^[6, 7]^表明，在肺癌患者中，*EGFR*基因突变与*KRAS*基因突变不能共存，有*KRAS*突变的患者对EGFR-TKI不敏感。一项临床研究^[8]^结果指出，*KRAS*突变患者对TKI的临床反应率不足3%。这提示*KRAS*突变可能与EGFR-TKI原发性耐药有关。Pao等^[6]^研究的60例肺腺癌患者中，38例对TKI耐药的患者中有9例存在*KRAS*突变，但无一例存在*EGFR*突变，17例*EGFR*突变患者服用TKI均有效，而9例*KRAS*突变患者服用TKI均无效。这为*KRAS*突变与TKI的原发耐药相关提供了临床依据。BR21研究^[9]^中接受厄洛替尼治疗的*KRAS*基因突变患者，与安慰剂组相比，生存期明显缩短，提示*KRAS*突变可以导致EGFR-TKI原发耐药，是其治疗的反指征。

### 2.2 EGFR耐药突变

NSCLC患者中有大约5%具有外显子20的插入或复制突变，此突变与外显子19的缺失突变和外显子21的L858R突变不同，它对EGFR-TKI的治疗不敏感，与EGFR-TKI的原发耐药有关。此外，有研究^[10]^发现在某些有较高肺癌发生率的家族中，发生于EGFR激酶结构域的外显子20的T790M突变，即790位点的苏氨酸被蛋氨酸取代，也与EGFR-TKI的原发耐药有关。但这一突变主要在NSCLC患者对TKI的获得性耐药中起主导作用（约占50%）。除T790M突变外，EGFR-TKI的原发耐药也可能与EGFR的其他二次突变有关，例如D761Y突变和E709A突变，它们常与EGFR的药敏突变同时发生，导致TKI的原发耐药。体外研究^[11]^亦证实*EGFR*双突变体与单突变体相比，对EGFR-TKI的敏感性要差。

### 2.3 PI3K/AKT信号通路的激活

EGFR的药敏突变导致EGFR-TKI耐药的原因，可能是存在影响下游信号的基因突变。例如，*EGFR*突变的细胞中PTEN表达下调或缺失时，使得PI3K-AKT过度激活，AKT的过度表达将抵抗凋亡，进而产生对EGFR-TKI的原发性耐药。有实验室研究^[12]^发现，PTEN的表达下调或缺失也与NSCLC的获得性耐药有关，但尚没有临床证据的支持。同时，研究^[13]^发现PIK3CA（PI3K的P110α催化亚单位）突变在有*EGFR*突变的日本肺癌患者中发生率为1.3%，而在没有*EGFR*突变的肺癌患者发生率为2.1%。体外研究^[14]^已经证实，一个组成性活化PI3K的点突变E545K能产生EGFR-TKI耐药。

### 2.4 胰岛素样生长因子1受体（insulin like growth factor 1 receptor, IGF1）介导的信号通路

IGF1R信号途径与EGFR信号途径的交互作用也是造成*EGFR*突变细胞耐药的机制。例如，对于*EGFR*突变的NSCLC细胞系同时使用厄洛替尼和IGF1R抑制剂时，可以诱导细胞凋亡的发生以及细胞周期停滞，但各自单独应用时，则只能导致细胞周期停滞，不会诱导细胞凋亡。可能是因为厄洛替尼虽然能持续下调EGFR和ERK磷酸化水平，但由IGF1R介导的AKT活化仍然能起抵抗凋亡的作用。此外，Sharma等^[15]^研究发现，*EGFR*突变的NSCLC患者使用EGFR-TKI治疗后，体内出现了敏感与耐药混合的细胞亚群，其中耐药细胞染色质的特殊状态是由IGF1R介导的信号途径和组蛋白脱甲基酶所调节的。同时，有研究^[16]^指出IGF1R介导的信号通路也是造成EGFR-TKI获得性耐药的机制。但是，这一结论仅得到了体外实验的证实，尚未有临床证据证明其与获得性耐药有关。

### 2.5 NF-κB信号途径的激活

Bivona等^[17]^发现核转录因子kappa B（nuclear factor-κB, NF-κB）信号途径的激活也会引起NSCLC对厄洛替尼的原发耐药。在具有*EGFR*突变的肺癌模型中，使用基因学或药理学方法对NF-κB信号通路进行抑制后可以增加突变肿瘤细胞对厄洛替尼治疗的敏感性。同样，NF-κB抑制因子IκB的低表达是使用厄洛替尼治疗无T790M突变的NSCLC患者不良预后的预测指标。这些结论一致表明NF-κB信号途径的过度激活可能引起*EGFR*突变的NSCLC患者对EGFR-TKI的原发性耐药。

### 2.6 *EML4-ALK*融合基因突变

研究^[18]^发现，*EML4-ALK*融合基因在动物体内外实验中均有明显的致瘤活性，在NSCLC的发生发展过程中起着重要作用，*EML4-ALK*融合基因突变主要发生于年轻、不吸烟的肺腺癌患者中，病理类型一般为腺泡型或实体腺癌伴粘液分泌型，与*EGFR*、*KRAS*突变不共存。Shaw等^[19]^发现在很少或不吸烟且没有*EGFR*突变的NSCLC患者中，有33%的患者可检测出*EML4-ALK*融合基因；同时，他们还对53例NSCLC患者进行EGFR-TKI药物治疗，其中疗效明显的19例患者均不存在*EML4-ALK*融合基因，而对EGFR-TKI耐药的34例患者中有29%可检测出该融合基因。因此，*EML4-ALK*融合基因可能是EGFR-TKI原发性耐药的重要机制。

### 2.7 *BRAF*基因突变

*BRAF*基因是位于EGFR信号通路下游的信号分子，BRAF蛋白介导RAS与MAPK结合，调节肿瘤细胞增殖、分化和凋亡。约2%-3%的NSCLC患者存在BRAF突变，其中最常见的是V600E^[20]^。有研究^[21]^表明，*BRAF*突变主要发生于肺腺癌患者，与*KRAS*和*EGFR*基因突变不共存。隐含*BRAF*基因V600E的NSCLC患者对MEK抑制剂PD0325901敏感，但对EGFR-TKI耐药。但由于*BRAF*在NSCLC中突变率较低，尚需大样本研究证实此突变是否与EGFR-TKI的原发性耐药有关。

### 2.8 人表皮生长因子受体-2（human epidermal growth factor receptor-2, HER-2）突变

HER-2蛋白同EGFR一样，也是表皮生长因子受体HER家族的成员之一，可以与其他HER家族成员形成同源或异源二聚体，并在NSCLC发生发展过程中起重要作用。*HER-2*基因突变常见于年轻不吸烟的亚裔女性，病理类型多为腺癌，与*EGFR*及*KRAS*突变不共存。约2%的NSCLC患者存在*HER-2*基因突变^[22]^。此突变使得受体持续激活，从而导致肿瘤细胞不断增殖、转移。体外研究^[23]^已经证实*HER-2*突变的NSCLC细胞株对EGFR-TKI耐药。Han等^[24]^的研究发现4例*HER-2*突变的NSCLC患者中无一例对吉非替尼有反应，从而为*HER-2*基因突变可能导致EGFR-TKI原发耐药提供了临床依据。

### 2.9 c-Met/肝细胞生长因子（hepatocyte growth factor, HGF）信号通路

*c-Met*基因扩增和过度表达参与了NSCLC对EGFR-TKI的原发性和获得性耐药这两种机制。对于有EGFR激活突变的NSCLC腺癌细胞系，*c-Met*基因扩增和过度表达可使得自分泌信号通过c-Met/HGF袢传导，导致PI3K/AKT信号途径传导恢复，而不依赖于EGFR或ERBB3的激活，从而产生对EGFR-TKI的原发性耐药^[25]^。同时，*c-Met*基因扩增也是20%NSCLC患者获得性耐药的重要机制^[26]^。肝细胞生长因子受体（hepatocyte growth factor, HGF）表达增加也会过度激活MET介导的PI3K/AKT通路，降低EGFR-TKI对这种信号级联反应的抑制。与获得性耐药作用机制不同，原发性耐药主要是通过GRB2相关结合蛋白1增加MET的HGF活化，而不是ERBB3的作用^[27]^。

### 2.10 成纤维细胞生长因子（fibroblast growth factor, FGF）-成纤维细胞生长因子受体（fibroblast growth factor receptor, FGFR）信号途径

特异性的FGF及FGFR是NSCLC肿瘤细胞自分泌信号通路的重要组成部分。Marek^[28]^和Kuhn^[29]^等发现反义RNA、RNA干扰技术、中和FGF2抗体以及FGFR-TKI等可以抑制NSCLC细胞系的增殖及肿瘤生长。说明FGF和FGFR的共表达为某些NSCLC细胞系提供了自分泌生长信号通路。此通路也被证实可以介导EGFR-TKI耐药。FGF-FGFR信号通路主要存在于肺鳞癌和大细胞癌中，使肿瘤细胞更倾向于向间充质状态分化^[28, 30]^。这与对EGFR-TKI敏感的肺腺癌及支气管肺泡癌原发肿瘤或细胞系的分化状态的多样化明显不同。Thomson等^[30]^还发现细胞内信号转导向FGFR及血小板源性生长因子受体（platelet drived growth factor receptor, PDGFR）介导的信号通路的转变与上皮细胞-间质转化（epithelial-mesenchymal transition, EMT）的发生同步。而EMT现象已被研究证实与EGFR-TKI耐药相关^[31]^。

### 2.11 EMT现象

EMT参与了胚胎发育过程中的器官塑性，是一种重要的自然生理现象。研究^[32]^发现，很多上皮来源的肿瘤都存在EMT，如肺癌、乳腺癌等。上皮细胞在失去上皮特征而重获间质特征以后，在侵袭、抗凋亡及转移能力方面都有很大提高。转化生长因子β1（transforming growth factor-β1, TGFβ1）、FGF、HGF、PDGF等均可能是EMT的诱导因子。同时，参与诱导EMT的信号通路（如Src、MAPK及PI3K等）均在EGFR-TKI耐药中发挥作用。研究认为，EGFR-TKI的原发性耐药株通常表现为上皮基因表达下调，出现间质细胞表型，即EMT现象。Coldren等^[31]^也通过研究证实EMT是NSCLC对EGFR-TKI原发耐药的机制之一。同时，EMT也已在实验室细胞模型中被证实与EGFR-TKI获得性耐药相关^[33, 34]^。最近，有研究^[35]^发现EMT确实存在于获得性耐药的*EGFR*突变的NSCLC患者中。但耐药肿瘤组织中的间质样细胞是在TKI治疗前即存在，还是在TKI治疗过程中诱导发生尚不明确。

## 3 获得性耐药

虽然在*EGFR*突变的NSCLC患者中吉非替尼和厄洛替尼可以起到很好的疗效，但大部分患者在治疗6个月-12个月内会发生获得性耐药。目前对EGFR-TKI获得性耐药机制的认识主要有以下几个方面。

### 3.1 *EGFR*二次点突变

*EGFR*的二次点突变可能是在EGFR-TKI治疗过程中产生的，但也有大量证据支持此突变是在开始TKI治疗前即存在，只是通过吉非替尼或厄洛替尼的作用被筛选出了耐药克隆。T790M突变是NSCLC患者中最常见的EGFR-TKI获得性耐药机制，其突变位点在20外显子，即酪氨酸激酶活化域的790位苏氨酸残基被蛋氨酸取代。TKI获得性耐药的*EGFR*突变患者中有50%存在T790M突变^[36, 37]^。其导致耐药的可能机制：①790位点的苏氨酸（T）被一个较大体积的蛋氨酸（M）替代，出现位阻效应，减弱了EGFR与ATP口袋中药物的结合力。蛋氨酸的一条较大的氨基酸侧链构成的空间位阻，阻止了TKI和EGFR酪氨酸激酶催化域中的Mg-ATP位点的结合，但对ATP和酪氨酸激酶的结合没有影响，激酶可以继续磷酸化。②T790M突变使EGFR与ATP的亲和力增加了至少10倍，恢复到野生型EGFR水平，ATP可以完全取代EGFR-TKI与EGFR结合。近年来又发现了3个与获得性耐药有关的EGFR第二位点突变，包括D761Y（外显子19）、T854A（外显子21激活环）^[38]^和L747S（外显子19）^[39]^。这些突变的发生率较低，三者发生率总和不到5%。其中，D761位于α-C的螺旋段，突变成酪氨酸后可能会影响盐桥的形成，且干扰受体的催化区；T854是与药物接触的氨基酸，突变成更小的丙氨酸可能会增加特异性口袋的尺寸，减弱甚至抵消与EGFR-TKI的结合力；L747位于β3链和α-C螺旋结构之间环区起始段，是调控受体活性构象平衡的重要氨基酸。

### 3.2 *MET*基因扩增

MET是HGF的受体，编码HGF酪氨酸激酶受体的跨膜区，与肿瘤的侵袭、转移和扩增有关。2007年，Engelman等^[40]^首次提出原癌基因*MET*的扩増是EGFR-TKI的一种耐药机制；他们在构造吉非替尼耐药细胞株模型时发现这种耐药是由*MET*基因扩增引起的；有22%的EGFR-TKI耐药患者的肿瘤组织中存在*MET*基因扩增。*MET*原癌基因扩增存在于20%的TKI获得性耐药的NSCLC患者中，其中有近一半的患者同时具有T790M突变。MET扩增通过激活ERBB3-PI3K信号途径来持续激活下游的信号通路，从而避开EGFR-TKI的靶点-EGFR，导致NSCLC对TKI产生耐药。MET的配体HGF除了可以导致原发性耐药以外，也能引起TKI的获得性耐药。Turke等^[27]^通过检测并对比27例用药前后的NSCLC患者的肿瘤组织，发现有16例患者在经过TKI治疗后HGF增多。Guix等^[41]^研究发现HGF通过选择性扩增*MET*基因过表达的克隆而发挥对EGFR-TKI的耐药作用。

### 3.3 *EGFR*突变基因丢失或拷贝数下降

Tabara等^[42]^研究发现*EGFR*突变基因丢失或拷贝数下降可能会引起EGFR-TKI的获得性耐药。在建立的厄洛替尼耐药细胞系中，*EGFR*激活突变（外显子19 delE746-A750或外显子21 L858R）的基因拷贝完全或部分丢失。而野生型*EGFR*基因拷贝数没有变化。因此，推测*EGFR*突变基因丢失或拷贝数下降可能会导致TKI的获得性耐药。

### 3.4

NSCLC向小细胞肺癌（small cell lung cancer, SCLC）的组织学转变equist等^[35]^研究报道，在37例EGFR-TKI耐药的NSCLC肿瘤组织中有5例出现了向SCLC组织学类型的转变，促使这一转变的分子学改变尚不清楚，但此组织学转变在79例未经EGFR-TKI治疗的NSCLC患者的肿瘤组织中未发现。

### 3.5 其他机制

*EGFR* T790M突变和*MET*基因扩增是NSCLC患者EGFR-TKI获得性耐药的主要机制，占60%左右。而其他40%获得性耐药的原因还在积极探索中。如前所述，PTEN表达下调或缺失、IGF1R介导的信号通路及EMT等可能参与了EGFR-TKI原发性和获得性耐药两种机制。其他可能导致获得性耐药的机制尚有：①研究^[43]^报道mTOR与EGFR-TKI获得性耐药有关，mTOR通路被阻断后，肿瘤生长会受到干扰，可能是因为EGFR-TKI对核糖体p70S6激酶的活化不起作用；②ATP结合盒式转运蛋白（ATP binding cassette, ABC）的药泵激活后可将药物泵到细胞外。ABCG2蛋白突变后可以把TKI泵到细胞膜外，从而降低肿瘤细胞内TKI的药物浓度，产生耐药^[44]^。

## 4 结语

EGFR-TKI靶向治疗相对于传统化疗具有更大的优势，已成为晚期NSCLC的有效治疗手段。从基因学角度寻找适合靶向治疗的患者，能达到更好的治疗效果。然而，原发和获得性耐药现象不可避免地发生使靶向治疗的应用产生了瓶颈，增加了临床上治疗肺癌的难度。但是研究者们对EGFR-TKI耐药机制的不断探索，能够帮助我们找到新的可以预测药物疗效并指导治疗方案的分子学标志物，从而使靶向药物的治疗效果进一步提高，并能更好地选择EGFR-TKI的治疗对象。同时，我们还可以尝试克服EGFR-TKI耐药的发生，从而给耐药患者带来重获治疗的希望。
